# Gene Expression Divergence is Coupled to Evolution of DNA Structure in Coding Regions

**DOI:** 10.1371/journal.pcbi.1002275

**Published:** 2011-11-17

**Authors:** Zhiming Dai, Xianhua Dai

**Affiliations:** School of Information Science and Technology, Sun Yat-Sen University, Guangzhou, China; Weizmann Institute of Science, Israel

## Abstract

Sequence changes in coding region and regulatory region of the gene itself (cis) determine most of gene expression divergence between closely related species. But gene expression divergence between yeast species is not correlated with evolution of primary nucleotide sequence. This indicates that other factors in cis direct gene expression divergence. Here, we studied the contribution of DNA three-dimensional structural evolution as cis to gene expression divergence. We found that the evolution of DNA structure in coding regions and gene expression divergence are correlated in yeast. Similar result was also observed between *Drosophila* species. DNA structure is associated with the binding of chromatin remodelers and histone modifiers to DNA sequences in coding regions, which influence RNA polymerase II occupancy that controls gene expression level. We also found that genes with similar DNA structures are involved in the same biological process and function. These results reveal the previously unappreciated roles of DNA structure as cis-effects in gene expression.

## Author Summary

The unique phenotype of each organism is partly determined by gene expression. Changes in gene expression are an important source of phenotypic variation, and can be caused by changes in regulatory and coding sequences of the gene itself (cis) and changes in regulatory factors (trans). The contribution of cis regulation to gene expression divergence between closely related species is much greater than that of trans regulation. However, evolution of primary nucleotide sequences is not correlated with gene expression divergence in yeast, suggesting that other factors in cis drive gene expression divergence. Here, we found that evolution of DNA structure in coding regions is coupled to gene expression divergence in yeast. We also found that DNA structure is associated with specific gene characteristics. Genes with similar DNA structures are involved in the same biological process and function. These results demonstrate the important roles of DNA structure in directing gene expression.

## Introduction

Proper control of gene expression is central for the unique phenotype of each organism. Phenotypic diversity can be generated through changes in gene expression. Divergence in gene expression of a specific gene between closely related species can result from sequence changes in its coding region and regulatory region (cis), or from changes in sequences or expression of its direct or indirect upstream regulators (trans). The binding of transcription factors (TFs) to sequence-specific sites in gene upstream regions plays a very important role in regulation of gene expression. Changes in TF-binding sequences and changes in abundance and binding domains of TFs can influence TF binding, which may cause variation in gene expression. The divergence of gene expression is also coupled to that of gene sequences in multicellular organisms [1]–[7]. In addition, as chromatin structure is critical for the regulation of gene expression, gene expression divergence between species correlates with divergence of nucleosomal organization [8], [9]. Nucleosome positioning is determined by cis effects (i.e. the intrinsic DNA sequence preference for nucleosome), and trans effects (e.g. chromatin modifiers).

The effects of cis and trans regulation on gene expression divergence can be measured by comparison of different strains of the same species [10], [11] and by analysis of hybrid species [12], [13]. Experiments on specific genes have revealed that the contribution of cis regulation to gene expression divergence between *Drosophila* species is much greater than that of trans regulation [14]. A genome-wide study on yeast species has also reproduced similar observation [15]. Cis-regulatory changes in gene expression are supposed to be driven by sequence mutations in TF binding sites or those in coding regions. However, most mutations in TF-binding sequences between yeast species have only little effect on gene expression divergence [16], though it cannot rule out the possibility that backup mechanisms exist among TF binding. Moreover, evolution of gene sequence in coding regions and gene expression divergence are not correlated in yeast [17]. These results leave open the question of what drive gene expression divergence in cis.

The three-dimensional structure of DNA, which reflects the physicochemical and conformational properties of DNA, is critical for the packaging of DNA in the cell [18]. The structure of DNA has been recognized to be important for protein-DNA recognition [19], [20]. Specific proteins-DNA interactions are fundamental to many biological processes, such as transcription, recombination, and DNA replication. DNA bending plays a role in the regulation of prokaryotic transcription [21]. DNA structure can be used as discriminatory information to identify core-promoter regions [22], [23]. Specific replication-related proteins show a preference to bind curved DNA sequences [24]. DNA curvature is also involved in the binding of recombination-related proteins [25].

A recent study has found that DNA structure in the human genome is more evolutionary constrained than the primary nucleotide sequence alone [26]. Moreover, the DNA structure-conserved regions correlate with non-coding regulatory elements, better than sequence-conserved regions identified solely on the basis of primary sequence [26]. These results indicate that DNA structure is important for regulation of gene expression. We presume that DNA structure is an ideal candidate for directing gene expression divergence in cis.

We evaluated DNA structure in terms of various physicochemical and conformational properties. We found that high levels of cis-driven gene expression divergence between yeast species correspond to high evolution rates of DNA structure in coding regions. This result also holds true between *Drosophila* species. The relationships of various types of structural evolution with gene expression divergence are conserved between yeast and *Drosophila*. We next investigated whether DNA structure is associated with gene characteristics. Genes that differ in DNA structure are distinguished by chromatin remodeler occupancy and histone modification levels, indicating that DNA structure influences gene expression by regulating the binding of chromatin regulators to DNA. Genes with similar DNA structures tend to belong to the same biological process and function.

## Results

### Evolution of Primary Nucleotide Sequence and Cis-Driven Gene Expression Divergence Are Not Correlated in Yeast

We examined the role of primary nucleotide sequence evolution in cis-driven gene expression divergence. Although a previous study has already found that gene expression divergence is not correlated with evolution of gene sequence in yeast [16], this result is confounded by the trans-effects in gene expression divergence. A recent study has designed a microarray to experimentally measure the relative contribution of cis and trans effects to gene expression divergence by using the hybrid of *Saccharomyces cerevisiae* and *Saccharomyces paradoxus* [15]. These valuable data allow for a direct examination of the contribution of primary nucleotide sequence evolution to cis-driven gene expression divergence.

First, we tested the relationship between sequence evolution in upstream regulatory regions and cis-effects to gene expression divergence. TF-binding sequences in promoter regions are the best-characterized elements that regulate gene expression. A previous study has analyzed the conservation of TF-binding sequences in promoters of closely related yeast species and identified the loss of TF-binding sites [27]. If mutation of TF-binding sequences influences gene expression divergence, genes with loss of TF-binding sites (i.e. whose promoters contain divergent sequence motifs) should show higher levels of cis-effects on gene expression divergence than genes without loss of TF-binding sites. However, genes with loss of TF-binding sites show relatively low levels of cis-effects on gene expression divergence (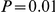, Mann-Whitney U-test; Figure S1A). Although changes of TF-binding sequences can significantly affect TF binding affinities which should be associated with changes in gene expression, backup mechanisms might compensate for the changes in TF-binding sequences which leads to the apparent little effect of loss of TF-binding sites on gene expression divergence. On the other hand, as yeast intergenic distances are relatively short, divergently oriented (i.e. reversely-oriented) gene pairs share a bi-directional cis-regulatory region in which TF-binding sequences might control the expression of both flanking genes [28]. If changes in TF-binding sequences have cis-effects on gene expression divergence, mutation of TF-binding sequences in a bi-directional cis-regulatory region might simultaneously influence gene expression divergence of both flanking genes. As a result, divergently oriented gene pairs should show higher similarity in cis-driven gene expression divergence levels than tandem or convergent gene pairs. However, we found that pair-wise differences in cis-effect levels for divergent gene pairs are comparable to those for tandem and convergent gene pairs (Figure S1B).

Second, we investigated into the contribution of sequence evolution in 3′ untranslated regions (UTR) to cis-driven gene expression divergence. Cis-regulatory elements in 3′ UTR are crucial for controlling RNA stability and expression. A previous study has calculated the evolutionary conservation of 3′ UTR cis-regulatory elements between closely related yeast species [29]. If mutation of 3′ UTR cis-regulatory elements influences gene expression divergence, genes with divergent 3′ UTR cis-regulatory sequence should show higher levels of cis-effects on gene expression divergence than genes with conserved 3′ UTR cis-regulatory sequences. However, the two classes of genes show comparable levels of cis-driven gene expression divergence (Figure S2).

Third, we examined the relationship between gene sequence evolution and cis-effects on gene expression divergence. In the measurement of contribution of cis effects to gene expression divergence [15], as both alleles of each gene are under the same nuclear environment (the same trans effects) in the hybrid of *S. cerevisiae* and *S. paradoxus*, differences in their expression reflect cis effects on gene expression divergence [15]. We defined the genes whose both alleles show significant difference in gene expression (above 2-fold) within the hybrid as genes with significant cis-effects to gene expression divergence. This is a stricter threshold compared to that (1.4-fold) in the original literature [15]. Initially, we found that though genes with significant cis-effects to gene expression divergence show higher gene sequence evolutionary rates between *S. cerevisiae* and *S. paradoxus* than the other genes, the statistical significance is rather weak (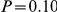 Mann-Whitney U-test; Figure S3; see Materials and Methods). This is consistent with the previous observation that evolution of gene sequence and gene expression divergence are not correlated in yeast [17]. Next, we examined whether cis-driven gene expression divergence is linked to codon bias. We found that genes with significant cis-effects to gene expression divergence and the other genes show similarity in codon bias divergence (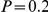, Mann-Whitney U-test; see Materials and Methods). This result suggests that cis-driven gene expression divergence between *S. cerevisiae* and *S. paradoxus* is not mainly caused by codon bias divergence.

### A Compendium of DNA Structural Properties

**Figure 1:**
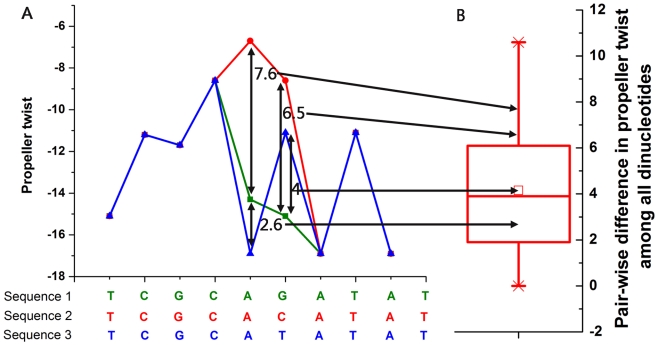
Significantly different changes in DNA structure by similar changes in DNA sequence. (A) Propeller twist patterns based on DNA dinucleotide (used as a measure of DNA structure, referred to here as the structural profile) and corresponding color-matched sequence alignments are shown. The sequence is divided into overlapping dinucleotide sequences. The corresponding propeller twist value for each dinucleotide was assigned to the first nucleotide of the dinucleotide. Sequence 1 is the reference sequence. We changed the base of the sixth position on the reference sequence and measured its effect on the structural profile. These changes were quantitatively measured by calculating the distance between structural profiles, where low values indicate similar structure profiles and large values indicate different structure profiles. Note that the single-base substitution causes changes in the two overlapping dinucleotides (i.e. AG and GA). (B) Box plot of values that correspond to pair-wise distance in structural parameters of propeller twist property among all DNA dinucleotides. The distance values in structural profiles in (A) were mapped to the box plot in (B). Single-base substitution causes significant change in DNA structure of Sequence 2 but only modest change in DNA structure of Sequence 3.

We have shown that genes with significant cis-effects to gene expression divergence and the other genes have comparable evolution rates of primary nucleotide sequence, indicating that evolution of primary nucleotide sequence in coding regions has little cis-effect on gene expression divergence in yeast. Although primary nucleotide sequences determine three-dimensional structures of DNA, and thus evolution rate of primary nucleotide sequences should correlate with evolutionary rate of DNA structures, this correlation is not complete. As similar changes in DNA sequence can cause significantly different changes in DNA structure (see Figure 1 for example), evolution of DNA structure might influence gene expression divergence. We thus asked whether genes with significant cis-effects to gene expression divergence show significant difference in evolution of DNA structure.

To test this possibility, we used 35 types of di- or trinucleotide DNA structural scales (Table S1), which were mainly collected in two references [23], [30]. The structural scales chosen in this study have been frequently used and have been extensively studied in previous literatures [31], [32]. These structural scales provide important information on the structure of DNA and capture structural properties that might be of importance for transcription. Each scale contains complementary information and provides a unique insight into the DNA structure (see Table S1 for more details about each of these structural scales). For the structural scales that have at least two different datasets, we used the most recently published dataset. The scales were classified into two types: conformational and thermodynamic [30]. The rationale for exploiting di- or trinucleotide properties is the widely accepted nearest neighbor model saying that DNA structure can be understood and caused largely by interactions between neighboring base pairs [33], [34]. This model is typically in the form of dinucleotide or trinucleotide scales. Each possible di- or trinucleotide and its reverse complement are assigned with a parametric value for a single structural property (Table S1). The origins of the parametric values are either derived from experimentally determined structures, or from simulated structures of a DNA helix or a DNA–protein complex.

**Figure 2:**
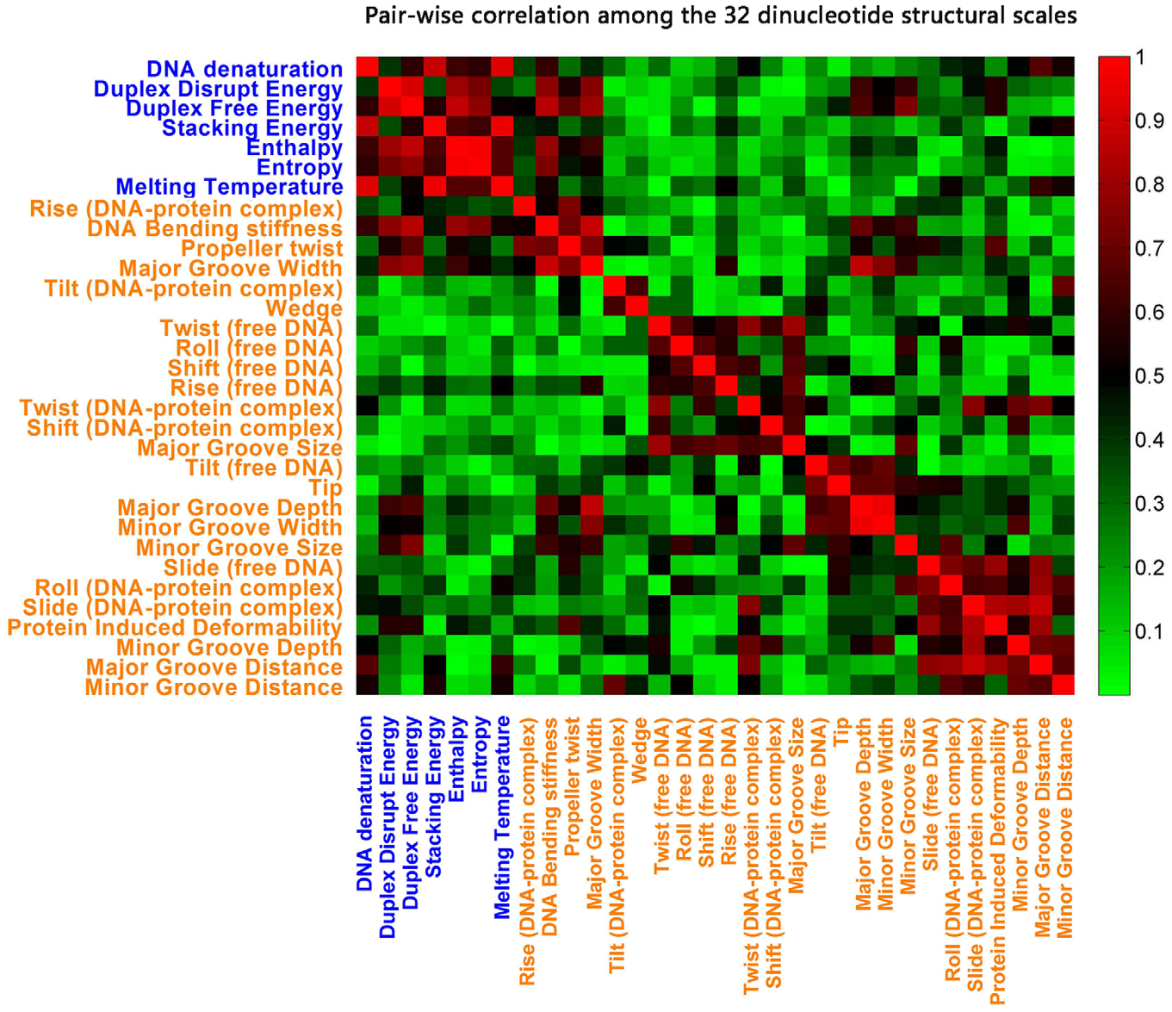
The pair-wise correlation among the 32 dinucleotide structural scales. Thermodynamic scales were in blue, while conformational scales were in orange. Each scale was represented by a vector of length 10 which contains the parametric values of dinucleotides. We calculated pair-wise Pearson correlation coefficients for the 32 scales (vectors), the absolute resulting values 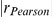 were shown. Red (green) indicates high (low) correlation. We classified the 32 scales into 5 clusters using K-means clustering based on the measure 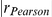.

In order to get insight into the different structural scales, we analyzed the structural data using principal component analysis (PCA) and clustering analysis. As most (32 out of 35) of the structural scales are based on dinucleotide, we performed the two analyses above on the dinucleotide structural scales. Considering that the dinucleotide and its reverse complement have the same parametric value for a single structural property, there are only 10 unique dinucleotides. We first performed a PCA calculating the 32 principal components for the 10 dinucleotides. Only the first 9 principal components (PCs) carry relevant information, roughly indicating that about this low number of scales is needed to represent all information of the complete set of 32 scales. As the first 5 PCs carry ∼88% of information (30%, 22%, 18%, 12%, and 6%), we next clustered the 32 scales into 5 classes using K-means clustering (Figure 2). Each scale was represented by a vector of length 10 which contains the parametric values of dinucleotides. We calculated pair-wise Pearson correlation coefficients for the 32 scales (vectors), and used the absolute resulting values 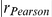 as the measure of the clustering. The absolute value of the correlation indicates whether two scales contain similar information. In Figure 2, it can be seen that all thermodynamic scales contain similar information. This is likely due to the fact that these thermodynamic scales are associated with the stability of DNA structure. Interestingly, the thermodynamic scales also contain similar information with some conformational scales, such as DNA bending stiffness and propeller twist. The rest of conformational scales are separated into four clusters. The most uncorrelated clusters (the lowest values in Figure 2) are the cluster containing all thermodynamic scales and the cluster containing twist (free DNA).

### Cis-Driven Gene Expression Divergence Is Associated with DNA Structural Evolution in Yeast Coding Regions

**Figure 3:**
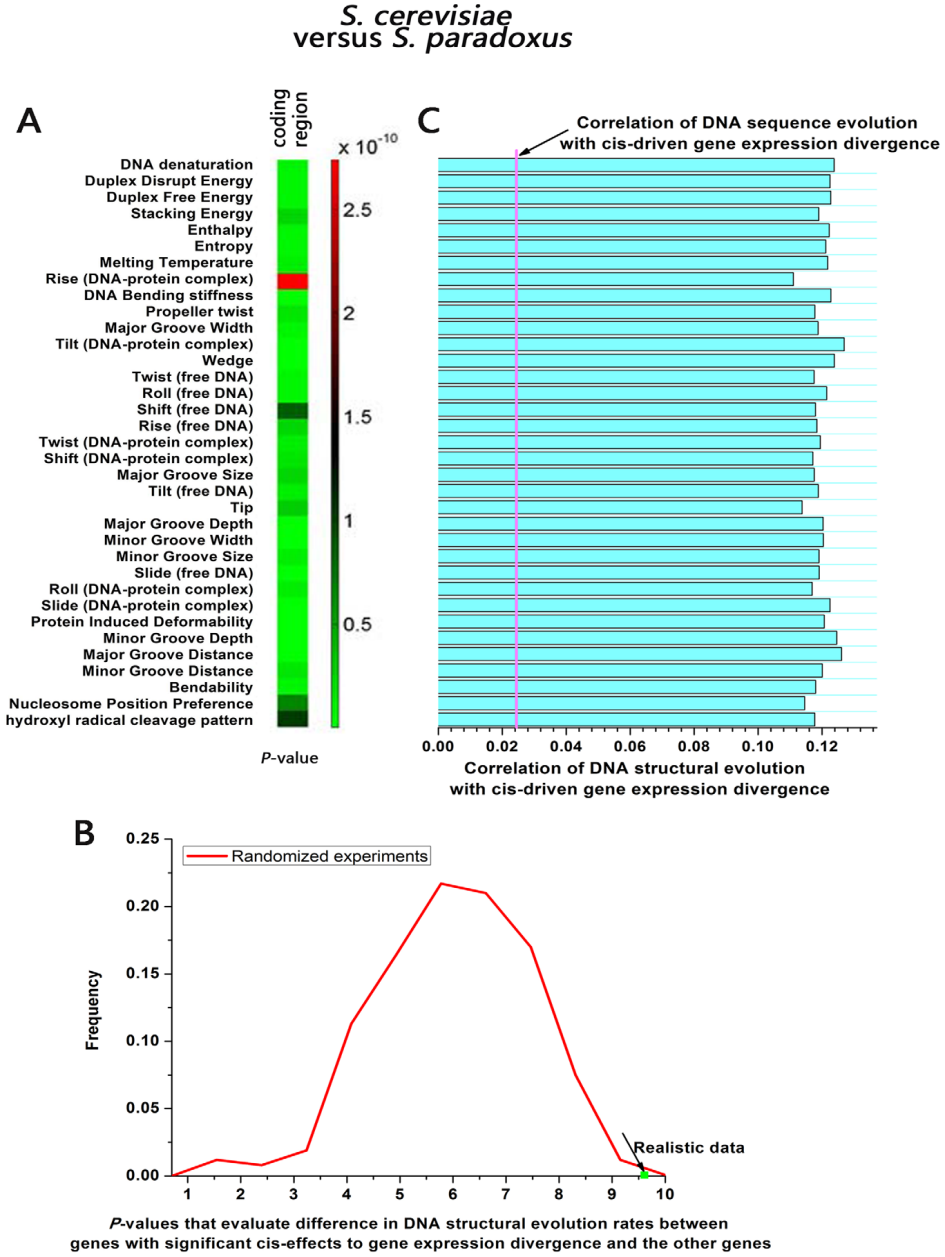
The correspondence of high levels of cis-driven gene expression divergence to high evolution rates of DNA structure. (A) In yeast, we compared the difference in 35 measures of DNA structural evolution rate (35 types of DNA structural scales) between genes with significant cis-effects to gene expression divergence and the other genes in their coding regions. Red (green) indicates high (low) *P*-values that evaluate the difference. Genes with significant cis-effects to gene expression divergence show significantly higher evolution rates of DNA structure in coding regions than the other genes in each of the 35 scales. (B) Distributions of *P*-values (Mann-Whitney U-test, −log10 transformed) that evaluate difference in DNA structural evolution rates in coding regions between genes with significant cis-effects to gene expression divergence and the other genes. The green dot is for the realistic parametric profile of rise (DNA-protein complex), while the red line depicts the distributions for 10,000 randomized experiments shuffling the parametric values. The structural scale rise (DNA-protein complex) was chosen because its statistical significance in (A) is the weakest. (C) Correlation of DNA structural evolution rate with cis-driven gene expression divergence is shown for each of the 35 DNA structural scales. Each bar represents the resulting Pearson correlation coefficients relative to that between primary nucleotide sequence evolution and cis-driven gene expression divergence (magenta line).

For each pair of orthologous genes between *S. cerevisiae* and *S. paradoxus*, we calculated DNA structural evolution rate for each of the 35 DNA structural scales (see Materials and Methods). Although DNA structural evolution rates show positive correlation with primary nucleotide sequence evolution rates, the correlation is not complete: The correlation coefficients range from 0.21 to 0.57 (Figure S4). As defined above, genes with significant cis-effects to gene expression divergence are the genes whose both alleles show significant difference in gene expression (above 2-fold) within the hybrid. Genes with significant cis-effects to gene expression divergence show significantly higher DNA structural evolution rates than the other genes in each of the 35 scales (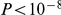, Mann-Whitney U-test, after Bonferroni correction for multiple testing, Figure 3A). In 5′ UTR and 3′ UTR, genes with significant cis-effects to gene expression divergence show comparable DNA structural evolution rates to those of the other genes in terms of each of the 35 scales (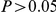, Mann-Whitney U-test). These results demonstrate that high levels of cis-driven gene expression divergence correspond to high evolution rates of DNA structure in coding regions.

The above correspondence of high cis-driven gene expression divergence with high evolution rates of all the 35 structural scales seems likely to be caused by evolution of primary nucleotide sequence. However, we have shown that genes with significant cis-effects to gene expression divergence show comparable gene sequence evolutionary rates with the other genes. These apparent discrepancies can be reconciled if different genes with significant cis-effects to gene expression divergence show higher evolution rates in different structural scales. As a result, genes with significant cis-effects to gene expression divergence as a whole show significantly higher evolution rates in all the structural scales. To test this possibility, we calculated the number of structural scales in which each gene with significant cis-effects to gene expression divergence shows significantly high evolution rates (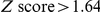, 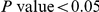). Indeed, we found that the resulting numbers range from 0 to 3 (Figure S5).

For each structural scale, we randomly shuffled the parametric values among the di- or trinucleotides. We generated 10,000 randomized profiles for each structural scale. We calculated DNA structural evolution rates in coding regions between orthologous genes as above based on these randomized profiles. If the correspondence between cis-driven gene expression divergence and DNA structural evolution observed above is not an artifact, the difference in DNA structural evolution rates between genes with significant cis-effects to gene expression divergence and the other genes should be more statistically significant than those based on the randomized structural profiles. For each structural scale, genes with significant cis-effects to gene expression divergence show higher DNA structural evolution rates in some of these shuffled profiles, but lower or comparable evolution rates in the other shuffled profiles. For each structural scale, most of the statistical significances (regardless of higher or lower evolution rates that genes with significant cis-effects to gene expression divergence show) in randomized experiments are weaker than that on the realistic profile (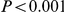, see Figure 3B for one example structural scale).

We next quantitatively evaluated the contribution of DNA structural evolution to gene expression divergence compared with that of primary nucleotide sequence evolution in coding regions. We calculated the correlation of primary nucleotide sequence evolution rate with cis-driven gene expression divergence (Pearson correlation coefficient, 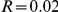). For each DNA structural scale, we calculated the correlation of its structural evolution rate with cis-driven gene expression divergence. We used the resulting correlation coefficients to represent the contribution of DNA structural evolution or primary nucleotide sequence evolution to cis-driven gene expression divergence. The correlation coefficients for DNA structural evolution are significantly higher than that for evolution of primary nucleotide sequence (Figure 3C). Moreover, when using partial correlation to control evolution of primary nucleotide sequence, DNA structural evolution is still significantly correlated with cis-driven gene expression divergence (Figure S6; see Materials and Methods).

We sought to evaluate the total contribution of DNA structural evolution to cis-driven gene expression divergence. Restricting analysis to genes with significant cis-effects to gene expression divergence, a multiple linear regression of cis-driven gene expression divergence against DNA evolution rates of 35 structural scales without considering any other factors gave an 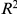 of 0.09 (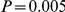), implying that about 9% of the variation of cis-driven gene expression divergence is attributable to DNA structural evolution. We also performed a linear regression of cis-driven gene expression divergence against primary nucleotide sequence evolution rates which gave an 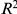 of 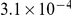. These results collectively demonstrate the significant association of DNA structural evolution with gene expression divergence relative to that of primary nucleotide sequence evolution. It is very interesting to explore what other factors in cis contribute to the variation of cis-driven gene expression divergence. Although we have found that genes with loss of TF-binding sites and genes with divergent 3′ UTR cis-regulatory sequences do not show significantly high cis-driven gene expression divergence (Figure S1, S2), it is very likely that divergence of unknown elements in promoters and 3′ UTR could be associated with cis-driven gene expression divergence.

As gene expression divergence data we used above were measured in a microarray [15], we examined whether the correspondence of cis-driven gene expression divergence to DNA structural evolution is an artifact of bias in microarray data. First, we examined the structural evolution of DNA sequences in the microarray probes. Changes in structural properties at the probe sequences might influence microarray hybridization and thus lead to apparent cis-driven gene expression divergence. We found that genes with significant cis-effects to gene expression divergence and the other genes show comparable DNA structural evolution rates in probe regions in terms of each of the 35 scales (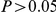, Mann-Whitney U-test, Figure S7; see Materials and Methods). Moreover, when restricting analysis to genes whose probe sequences have low structural evolution rates, genes with significant cis-effects to gene expression divergence still show significantly higher DNA structural evolution rates in coding regions than the other genes in each of the 35 scales (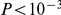, Mann-Whitney U-test, after Bonferroni correction, Figure S8). These results indicate that cis-driven expression divergence is not an artifact caused by DNA structural evolution in microarray probe regions. Second, we tested the relationship of cis-driven gene expression divergence with DNA structural evolution using gene expression divergence data between *S. cerevisiae* and *S. bayanus* measured in RNA-seq platform [35]. We found that genes with significant cis-effects to gene expression divergence show significantly higher DNA structural evolution rates in coding regions than the other genes in each of the 35 scales (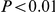, Mann-Whitney U-test, Figure S9). These results collectively indicate that the relationship of cis-driven gene expression divergence to DNA structural evolution is robust to the choice of experimental platforms.

### High Levels of Gene Expression Divergence Correspond to High DNA Structural Evolution Rates in *Drosophila* Coding Regions

We examined the relationship of gene expression divergence to DNA structural evolution in other species. Previous studies have revealed a significant positive correlation between evolution rate of gene sequence and gene expression divergence in *Drosophila* species [2], [4]. As different DNA sequences might have similar DNA structures [26], high evolution rates of primary nucleotide sequence do not always correspond to high evolution rates of DNA structure. The relationship between evolution of DNA structure and gene expression divergence in *Drosophila* species remains to be elucidated. Using gene expression divergence data in *Drosophila* [36], [37] and the 35 DNA structural scales above, we found that genes with significant cis effects on gene expression divergence also show significantly higher DNA structural evolution rates than the other genes (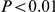, Mann-Whitney U-test, Figure S10). When normalizing DNA structural evolution rates by gene sequence evolution rates, genes with significant cis effects on gene expression divergence still show higher normalized DNA structural evolution rates than the other genes (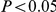, Mann-Whitney U-test, Figure S10), albeit with weaker statistical significance. Taken together, these results demonstrate that the relationship between DNA structural evolution and gene expression divergence is conserved between *Drosophila* and yeast species.

We further examined whether the relationships of 35 types of structural evolution with gene expression divergence are conserved. For each type of structural evolution, we used the above *P*-value from Mann-Whitney U-test, which was performed between genes with significant cis-effects to gene expression divergence and the other genes, to represent the degree of contribution of this type of structural evolution to gene expression divergence. The more significant the *P*-value is, the more the contribution is. We found that *S. cerevisiae-S. paradoxus* pair and *D. melanogaster-D. simulans* pair, *S. cerevisiae-S. paradoxus* pair and *D. melanogaster-D. sechellia* pair, *D. melanogaster-D. sechellia* pair and *D. melanogaster-D. simulans* pair show significant positive correlation in the contribution of structural evolution to gene expression divergence (Table S2). However, *S. cerevisiae-S. bayanus* pair shows no correlation with the other three pairs.

### High DNA Structural Evolution Rates Correspond to High Levels of Cis-driven Gene Expression Divergence

**Figure 4:**
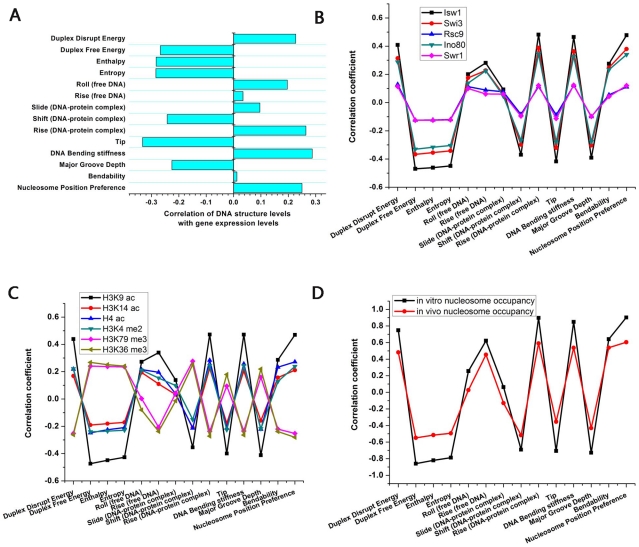
The correlation of DNA structural levels in coding regions with characteristic gene features. (A) DNA structure levels show significant correlation with gene expression levels in *S. cerevisiae*. Each bar represents the resulting Pearson correlation coefficients for the 14 DNA structural scales. 12 out of the 14 scales show significant correlation with gene expression levels. (B) DNA structure levels show significant correlation with chromatin remodeler occupancy in coding regions. The Pearson correlation coefficients are indicated for the 14 structural scales. (C) DNA structure levels show significant correlation with histone modification levels in coding regions. The Pearson correlation coefficients are indicated for the 14 structural scales. See Table S3 for the full result of all 25 histone modifications. (D) DNA structure levels show significant correlation with *in vitro* and *in vivo* nucleosome occupancy in coding regions. The Pearson correlation coefficients are indicated for the 14 structural scales.

We have shown that high levels of gene expression divergence correspond to high evolution rates of DNA structure, but whether the converse relationship holds true remains to be answered. In the following analysis, we focused on DNA structural evolution in coding regions between *S. cerevisiae* and *S. paradoxus*. We first identified cohort of genes for each DNA structural scale. Genes belong to the cohort of one DNA structural scale if they display significantly high evolution rates (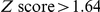, 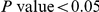) of the corresponding DNA structural scale in coding regions. In this way, we obtained 35 sets of cohorts. 14 out of the 35 gene cohorts show significantly higher cis-driven gene expression divergence than the other genes (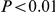, Mann-Whitney U-test, after Bonferroni correction; See Figure 4A for the list of the 14 structural scales). Considering only dinucleotide scales, we found that absolute values of pair-wise Pearson correlation coefficients among parametric values (i.e. profiles) of these significant dinucleotide scales are comparable to those among the other scales (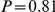, Mann-Whitney U-test), ruling out their potential redundancy in DNA structure. For these 14 DNA structural scales, their high structural evolution rates can cause high gene expression divergence. Whereas for the other DNA structural scales, though high gene expression divergence can be explained by their high structural evolution rates, other factors might limit the contribution of their structural evolution to gene expression divergence, which leads to the observation that their high evolution rates do not correspond to high gene expression divergence. In the following analysis, we focused on these 14 significant DNA structural scales.

### Gene Expression Level Is Correlated with DNA Structural Levels in *S. cerevisiae* Coding Regions

We investigated into the roles of DNA structure in gene expression in a single species. We have shown that evolution of DNA structure in coding regions is correlated with gene expression divergence. If this correlation is biologically meaningful, DNA structural levels in coding regions should also be correlated with gene expression levels in a single species. For each of the 14 significant DNA structural scales above, we calculated the structural profile in each coding region from DNA sequences (see Materials and Methods), and used the average value of the structural profile to represent the level of this structural scale in the coding region. We found that structural levels of 12 out of the 14 scales show significant correlation with gene expression levels (Pearson correlation coefficient, 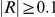,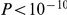, Figure 4A). Similar results were reproduced on gene transcription rate data and RNA polymerase II occupancy in coding regions (Figure S11), implying that most of these correlations are caused at the transcriptional level. 6 scales show significant positive correlation, while 6 scales show significant negative correlation (Figure 4A).

4 thermodynamic scales, including duplex disrupt energy, duplex free energy, enthalpy and entropy, show significant correlation with gene expression levels. As duplex disrupt energy is positively correlated with stability of DNA duplex and the other three scales is negatively correlated with stability of DNA duplex, these results indicate that stability of DNA duplex in coding regions is positively correlated with gene expression levels. It has been shown that RNA polymerase elongation tends to pause when the DNA duplex is unstable [38], [39]. The high stability of DNA duplex in coding regions should facilitate transcription elongation and raise mRNA gene expression level.

2 nucleosome-related scales, including DNA bending stiffness and nucleosome position preference, show significant positive correlation with gene expression levels. High values of DNA bending stiffness correspond to dinucleotides that will bend more easily, which facilitates the packaging of DNA into nucleosome. This result is consistent with previous observation that nucleosome occupancy within coding regions positively correlates with transcription level [40].

3 conformational scales, including rise (DNA-protein complex), roll (free DNA) and slide (DNA-protein complex), show significant positive correlation with gene expression levels. Following the definitions of the structural parameters in the EMBO workshop [41], these three scales are positively correlated with the distance between two successive base pairs. Maybe the increase in the distance between two successive base pairs in coding regions facilitates transcription. Another 2 scales, including shift (DNA-protein complex) and major groove depth, show significant negative correlation with gene expression levels. Shift (DNA-protein complex) could increase major groove depth which might influence gene expression.

### DNA Structural Levels Are Correlated with Chromosomal Features

We further investigated into how DNA structure influences gene expression. As chromatin remodeler occupancy and histone modification levels in coding regions influence gene expression, we examined the relationship of DNA structural levels with these two chromosomal features. First, we used genome-wide occupancy data for chromatin remodelers [42]. These data were measured with single-gene resolution based on microarray. We found that DNA structural levels show significant correlation with chromatin remodeler occupancy in coding regions (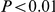, Figure 4B). Moreover, the directions of correlation are the same as those between structural levels and gene expression levels, indicating that these chromatin remodelers facilitate gene expression. Second, using available genome-wide histone modification data measured in microarray [43], [44], we found that DNA structural levels are also significantly correlated with histone modification levels (Figure 4C, Table S3). We also found that the bias of microarray probes on our observations is very limited (see Materials and Methods). DNA structure is critical for protein-DNA recognition. Difference in DNA structure might change the binding of chromatin remodelers and histone modifiers to DNA, leading to the difference in gene expression levels.

We next investigated into the relationship of DNA structural level with nucleosome occupancy. DNA sequence is an important determinant of nucleosome positioning which is critical for gene expression. A previous study has measured genome-wide *in vitro* nucleosome occupancy that is determined only by the intrinsic DNA sequence [45]. Sequences covered by high *in vitro* nucleosome occupancy have high sequence preference for nucleosome formation, while sequences covered by low *in vitro* nucleosome occupancy inhibit nucleosome formation. We found that DNA structural levels are significantly correlated with *in vitro* nucleosome occupancy in coding regions: some structural scales facilitate nucleosome formation, while others inhibit nucleosome formation (Figure 4D). We also found that DNA structural levels are also significantly correlated with *in vivo* nucleosome occupancy, though the correlations become weak (Figure 4D).

### Genes with Similar DNA Structures Are Involved in the Same Biological Process and Function

We asked whether DNA structure is linked to biological process and function. We have shown that DNA structure is associated with gene expression and chromatin regulators. As genes with similar gene co-expression patterns or genes regulated by similar regulators are known to be involved in similar biological processes and functions, we asked whether genes with similar DNA structural levels are involved in similar biological processes and functions. We tested this possibility using the 14 significant DNA structural scales above whose high evolution rates correspond to high gene expression divergence. As stated above, for each of the 14 DNA structural scales, we calculated the structural profile in each coding region from DNA sequences (see Materials and Methods), and used the average value of the structural profile to represent the level of this structural scale in the coding region. We sorted all yeast genes in ascending order based on the corresponding DNA structural levels for each DNA structural scale, and split them into five equal gene clusters. Genes in the same gene cluster have similar structural levels of the corresponding structural scale. We found that genes in the same gene cluster tend to belong to the same biological process or function as indicated by Gene Ontology [46] (see Table S4 for the full results of all structural scales). We found that genes in the same gene cluster are involved in diverse biological processes and functions, including those are regulatory or housekeeping. There is no gene cluster that is characterized only by regulatory or housekeeping processes. Different clusters also have some processes and functions in common. We also binned genes into different numbers (from 3 to 10) of equal groups based on their structural levels, respectively. Similar results that genes in the same gene cluster tend to belong to the same biological process or function could be reproduced, which indicates that our observation is robust to the choice of the numbers of gene clusters.

## Discussion

Cis-effects dominate gene expression divergence between yeast species. However, evolution of primary nucleotide sequences are not correlated with gene expression divergence, suggesting that other factors in cis drive gene expression divergence. Here, we used various physicochemical and conformational DNA properties to investigate into the relationship between evolution of DNA structure and gene expression divergence. We found that evolution of DNA structure in coding regions is coupled to gene expression divergence in yeast and in *Drosophila*. We also found that DNA structure in coding regions is associated with gene expression in a single species. DNA structure in coding regions is also associated with the binding of chromatin regulators to DNA that regulates gene expression, leading to the observed association between DNA structure and gene expression. These results highlight the important role of DNA structure as cis-effect in gene expression.

The evolution of both DNA sequence and structure in non-coding regulatory regions are not correlated with gene expression divergence. But gene expression has been thought to be mainly regulated by the regulatory elements in non-coding regions. These apparent discrepancies can be reconciled if backup mechanism exists in gene regulatory programs. A previous study has revealed that most genes in yeast are not affected when any TF is knocked out [47], indicative of redundant TFs which mask the TF knockout effect. As DNA binding sequences of TFs are usually short and degenerate, there might be multiple binding sequences for one specific TF in the regulatory region. This redundancy compensates for changes in TF-binding sequence, maybe leading to the apparent little effect of their changes on gene expression.

Although we found that DNA structure is associated with gene expression, the mechanisms of this relationship remain to be elucidated. We found that DNA structure is associated with distinct gene features. These results collectively reveal how DNA structure influences gene expression. We found that DNA structure is correlated with chromatin remodeler occupancy, histone modification levels and nucleosome occupancy. These results suggest that DNA structure influences the binding of chromatin remodelers and histone modifiers to DNA, and nucleosome positioning along DNA in coding regions. Chromatin remodeling, histone modification and nucleosome positioning could influence elongation of RNA polymerase II which controls gene expression. However, further experimental work will be required to more fully understand how DNA structure modulates gene expression.

## Materials and Methods

### Data Preparation

Yeast genome sequences and gene coordinate were downloaded from the *Saccharomyces* Genome Database (http://www.yeastgenome.org/). Yeast transcript coordinate data were taken from David et al. [48]. Orthologous genes between *S. cerevisiae* and *S. paradoxus* were taken from Wapinski et al. [49]. Orthologous genes and their sequences between *D. melanogaster* and *D. simulans* were taken from Heger et al. [50]. The relative contribution of cis and trans effects to gene expression divergence between *S. cerevisiae* and *S. paradoxus* were taken from Tirosh et al. [15]. As both alleles of each gene are under the same nuclear environment (the same trans effects) in the hybrid, differences in their expression reflect cis effects on expression divergence, whereas expression differences between the parental genes that disappear in the hybrid reflect trans effects. In the original literature, genes whose both alleles show >1.4-fold difference in gene expression within the hybrid were considered to have significant cis effects [15]. In this study, we set a stricter threshold and defined the genes whose both alleles show significant difference in gene expression (above 2-fold) within the hybrid as genes with significant cis-effects to gene expression divergence. Cis-driven gene expression divergence data between *S. cerevisiae* and *S. bayanus* were taken from Bullard et al. [35]. Genes with statistical significance 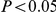 in the original literature were defined as genes with significant cis-effects to gene expression divergence. Gene expression and transcription rate data in *S. cerevisiae* were taken from Holstege et al. [51]. Gene expression divergence data between adults of *D. melanogaster* and *D. simulans* were taken from Ranz et al. [36]. Genes with statistical significance 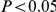 in the original literature were defined as genes with high levels of gene expression divergence. Gene expression divergence data between *D. melanogaster* and *D. sechellia* were taken from McManus et al. [37]. We used the same definition of genes with significance cis effects on gene expression divergence as that in the original literature [37].

The conservation of sequence motifs in promoters of closely related yeast species was analyzed and the loss of TF-binding sites was predicted by Doniger et al. [27]. We identified genes with loss of TF-binding sites (divergent) or without loss of TF-binding sites (conserved) in their promoters. This results in two gene clusters. Some genes have multiple TF-binding sites in promoter regions. Some binding sites in one promoter region might be conserved, ant the other binding sites in this promoter region might be divergent. Some genes thus might belong to two gene clusters simultaneously. We excluded genes shared by the two gene clusters for analysis. The evolutionary conservation of 3′ UTR cis-regulatory elements between yeast species were taken from Shalgi et al. [29]. 3′ UTR cis-regulatory sequences with significant conserved *P*-value 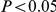 are considered to be conserved. As the method above, we identified genes with conserved 3′ UTR cis-regulatory elements and divergent 3′ UTR cis-regulatory elements, respectively.

Genome-wide *in vivo* and *in vitro* nucleosome occupancy data in *S. cerevisiae* were taken from Kaplan et al. [45]. We calculated the average *in vivo* and *in vitro* nucleosome occupancy in coding region for each gene, respectively. Genome-wide RNA polymerase II occupancy (RNA polymerase II subunit Rpo21) data in *S. cerevisiae* were taken from Venters et al. [42]. We calculated the average RNA polymerase II occupancy in coding region for each gene. Chromatin remodeler occupancy in coding regions was taken from Venters et al. [42]. Histone modification data were taken from ChromatinDB [43], a database of genome-wide histone modification patterns for *S. cerevisiae*. We added the histone modification data from Pokholok et al. [44], a total of 25 histone modifications. For each coding region, we calculated the average level for each histone modification.

### Calculation of Gene Sequence Evolutionary Rate

We performed the global alignment on gene sequences between orthologous genes. We used the rate of nonsynonymous substitutions (Ka) normalized by the rate of synonymous substitutions (Ks) as a measure of gene sequence evolutionary rate.

### Calculation of Codon Bias Divergence

We used the codon adaptation index (CAI) to indicate codon bias. We calculated CAI for each gene as a previous method [52]. For each pair of orthologous genes between *S. cerevisiae* and *S. paradoxus*, we calculated their absolute value of difference in CAI values, and defined the resulting value as its CAI divergence. We compared genes with significant cis-effects to gene expression divergence with the other genes in CAI divergence.

### Analyses of DNA Structural Scales

We used 35 types of conformational and thermodynamic DNA di- or trinucleotide structural scales, which were mainly collected by two references [23], [30], as measures of DNA structure. We normalized each of the 32 dinucleotide structural scales (their means are zero and standard deviations are one), and performed a PCA calculating the 32 principal components for the 10 dinucleotides. Each scale was represented by a vector of length 10 which contains the parametric values of dinucleotides. We calculated pair-wise Pearson correlation coefficients 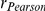 for the 32 scales (vectors), and classified the 32 scales into 5 clusters using K-means clustering based on the measure 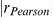.

### Calculation of DNA Structural Evolutionary Rate

For a DNA region, the sequence is divided into overlapping di- or trinucleotide sequences. Structural profiles from DNA sequences are calculated for each structural scale (except for hydroxyl radical cleavage pattern) as follows: The corresponding parametric value for each di- or trinucleotide was assigned to the first nucleotide of the di- or trinucleotide. In this way, the nucleotide sequence is converted into a sequence of numbers (i.e., a numerical profile). For hydroxyl radical cleavage intensity data, structural profiles are calculated as the reference where the data was published [53]. The hydroxyl radical cleavage intensity data are assigned to each nucleotide in each trinucleotide sequence. Note that the three nucleotides in each trinucleotide sequence have different values of hydroxyl radical cleavage intensity. As each nucleotide (except for the two terminal nucleotides at each end of the DNA region) is covered by three overlapping trinucleotide sequences, it has three values of hydroxyl radical cleavage intensity (one for each trinucleotide). The three values are averaged to produce hydroxyl radical cleavage intensity for each nucleotide. In this way, the nucleotide sequence is converted into a sequence of numbers (i.e., a numerical profile). For each pair of orthologous genes, we calculated the Euclidean distance of structural profiles after pairwise alignments on gene sequences between orthologous genes. We considered the resulting Euclidean distance normalized by the length of coding region as a measure of evolution rate of DNA structure. In this way, there were 35 measures of structural evolutionary rate for each pair of orthologous genes. We also calculated structural evolutionary rates for 5′ UTR and 3′ UTR for yeast species.

### Partial Correlation

Partial correlation can measure the degree of association between two variables with the effect of controlling variables removed. 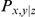 indicates the partial correlation between 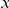 and 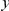 when controlling 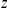. It is defined as:
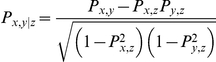
Where 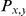 is the correlation between x and y. We calculated the partial correlation between DNA structural evolution rates and cis-driven gene expression divergence when controlling primary nucleotide sequence evolution rates.

### Evaluation of DNA Structural Bias in Microarray Probes

The DNA structural evolution rates in microarray probes which were used to measure gene expression divergence are calculated as follows. For each probe, we profiled the values of each specific structural scale versus its sequence positions, and called this graph its structural profile of this structural scale. For each pair of orthologous genes, we calculated the Euclidean distance between structural profiles of their two probes, and used the resulting values normalized by the length of the probe as a measure of evolution rate of DNA structure. For orthologous genes with more than one pair of probes, we calculated the Euclidean distance normalized by the length of the probe for each pair of probes, and used the average resulting distance value as a measure of DNA structural evolution rate. In this way, there were 35 measures of structural evolutionary rate in probe regions for each pair of orthologous genes.

To evaluate the microarray probe bias on the measurement of chromatin remodeler occupancy, we calculated for each coding region the average structural value of each structural scale across its coding regions after excluding the sequences of its microarray probe. The resulting DNA structure values are still significantly correlated with chromatin remodeler occupancy (data not shown).

For each probe in microarray that were used to measure histone modification level, we calculated the average structural value of each structural scale across its sequence positions. We found that histone modification levels are weakly correlated with the DNA structures in probe regions (Pearson correlation coefficients, 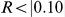), suggesting that the bias of probes in histone modification level is very limited.

## Supporting Information

Figure S1 The relationship between changes in primary nucleotide sequences of 5′ UTR and cis-driven gene expression divergence. (A) Box plot of average values that correspond to levels of cis-effects on gene expression divergence are shown for genes with loss of TF-binding sites and genes without loss of TF-binding sites. (B) Box plot of average values that correspond to absolute values of pair-wise difference in levels of cis-effects to gene expression divergence are shown for divergent gene pairs and the other gene pairs. Values were normalized using the function *zscore* in Matlab, such that their means are zero and standard deviations are one.
(JPG)

Figure S2 The relationship between changes in primary nucleotide sequences of 3′ UTR and gene expression divergence. Box plot of average values that correspond to levels of cis-effects on gene expression divergence are shown for genes whose 3′ UTR cis-regulatory sequences are less conserved (divergent) and genes with conserved 3′ UTR cis-regulatory sequences. Values were normalized using the function *zscore* in Matlab, such that their means are zero and standard deviations are one.
(JPG)

Figure S3 The relationship between changes in primary nucleotide sequences of coding regions and gene expression divergence. Box plot of average values that correspond to gene sequence evolutionary rates are shown for genes with significant cis-effects to gene expression divergence and the other genes. Values were normalized using the function *zscore* in Matlab, such that their means are zero and standard deviations are one. We performed the global alignment on orthologous gene sequences between *S. cerevisiae* and *S. paradoxus*, and used the rate of nonsynonymous substitutions (Ka) normalized by the rate of synonymous substitutions (Ks) as a measure of gene sequence evolutionary rate.
(JPG)

Figure S4 The correlation of DNA structural evolution rates with primary nucleotide sequence evolution rate. For each pair of orthologous genes between *S. cerevisiae* and *S. paradoxus*, we calculated gene sequence evolutionary rates and DNA structural evolution rate for each of the 35 DNA structural scales. The Pearson correlation coefficient between sequence evolutionary rates and structural evolution rates is shown for each of the 35 structural scales.
(JPG)

Figure S5The number of structural scales in which each of the genes with significant cis-effects to gene expression divergence shows significantly high evolution rates (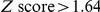, 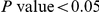). The distribution of the numbers is shown.(JPG)

Figure S6 Partial correlation of DNA structural evolution rate with cis-driven gene expression divergence is shown for each of the 35 DNA structural scales when controlling primary nucleotide sequence evolution rates. Each bar represents the resulting partial correlation coefficients.
(JPG)

Figure S7 The relationship of cis-driven gene expression divergence between *S.cerevisiae* and *S. paradoxus* with DNA structural evolution in microarray probe regions. We compared the difference in evolution rates of 35 DNA structural scales in microarray probe regions between genes with significant cis-effects to gene expression divergence and the other genes. *P*-values were calculated through Mann-Whitney U-test, and are shown for the 35 DNA structural scales. Red (green) indicates high (low) *P*-values that evaluate the difference.
(JPG)

Figure S8 The relationship of cis-driven gene expression divergence between *S. cerevisiae* and *S. paradoxus* with DNA structural evolution when restricting analysis to genes whose probe sequences have low structural evolution rates (the 50% percentile). For each of the 35 DNA structural scales, we excluded genes whose probe sequences have high structural evolution rates (the 50% percentile), and compared the difference in DNA structural evolution rate between genes with significant cis-effects to gene expression divergence and the other genes in their coding regions. *P*-values were calculated through Mann-Whitney U-test, and are shown for the 35 DNA structural scales. Red (green) indicates high (low) *P*-values that evaluate the difference.
(JPG)

Figure S9 The relationship of cis-driven gene expression divergence between *S. cerevisiae* and *S. bayanus* with DNA structural evolution. We compared the difference in evolution rates of 35 DNA structural scales between genes with significant cis-effects to gene expression divergence and the other genes in their coding regions. *P*-values were calculated through Mann-Whitney U-test, and are shown for the 35 DNA structural scales. Red (green) indicates high (low) *P*-values that evaluate the difference.
(JPG)

Figure S10 The relationship of cis-driven gene expression divergence in *Drosophila* species with DNA structural evolution. We compared the difference in evolution rates of 35 DNA structural scales between genes with significant cis-effects to gene expression divergence and the other genes in their coding regions. Comparison was also performed after normalizing DNA structural evolution rates by gene sequence evolution rates. *P*-values were calculated through Mann-Whitney U-test, and are shown for the 35 DNA structural scales. Red (green) indicates high (low) *P*-values that evaluate the difference.
(JPG)

Figure S11The correlation of DNA structural levels in coding regions with transcription rates and RNA polymerase II occupancy in coding regions. Each bar represents the resulting Pearson correlation coefficients for the 14 DNA structural scales. 12 out of the 14 scales show significant correlation with these two features (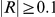,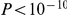).(JPG)

Table S1 List of dinucleotide/trinucleotide DNA structural scales and their corresponding parameters.
(XLS)

Table S2 The correlation in the contribution of structural evolution to gene expression divergence among Drosophila and yeast species. For each type of structural evolution, we used the *P*-value from Mann-Whitney U-test, which was performed between genes with significant cis-effects to gene expression divergence and the other genes, to represent the degree of contribution of this type of structural evolution to gene expression divergence.
(XLS)

Table S3 The correlation of DNA structural levels with histone modification levels in coding regions. The Pearson correlation coefficients are shown.
(XLS)

Table S4 The gene clusters which are clustered based on their DNA structural levels in coding regions. For each of the 14 structural scales, all genes are clustered into 5 groups based on their corresponding DNA structural levels, respectively. Biological processes and functions that the each gene cluster belongs to are shown. *P*-values for Gene Ontology terms were derived using ‘GO term finder’ at the Saccharomyces Genome Database.
(XLS)
