# Supplementary figures and images for: Gene Expression Divergence is Coupled to Evolution of DNA Structure in Coding Regions
Source: PLoS Comput Biol. 2011 Nov 17;7(11):e1002275. doi: 10.1371/journal.pcbi.1002275 (PMC3219629; doi:10.1371/journal.pcbi.1002275)

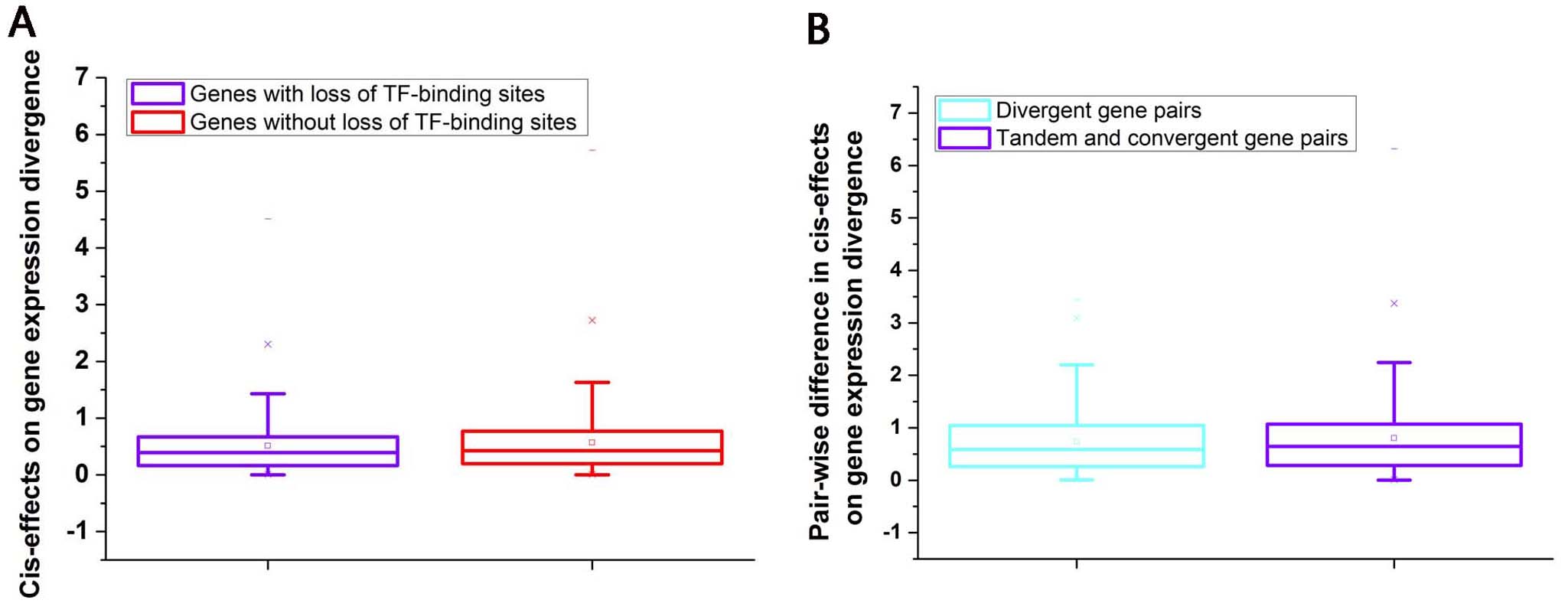

Supplement: Figure S1 — The relationship between changes in primary nucleotide sequences of 5′ UTR and cis-driven gene expression divergence. (A) Box plot of average values that correspond to levels of cis-effects on gene expression divergence are shown for genes with loss of TF-binding sites and genes without loss of TF-binding sites. (B) Box plot of average values that correspond to absolute values of pair-wise difference in levels of cis-effects to gene expression divergence are shown for divergent gene pairs and the other gene pairs. Values were normalized using the function zscore in Matlab, such that their means are zero and standard deviations are one. (JPG) [file pcbi.1002275.s001.jpg]

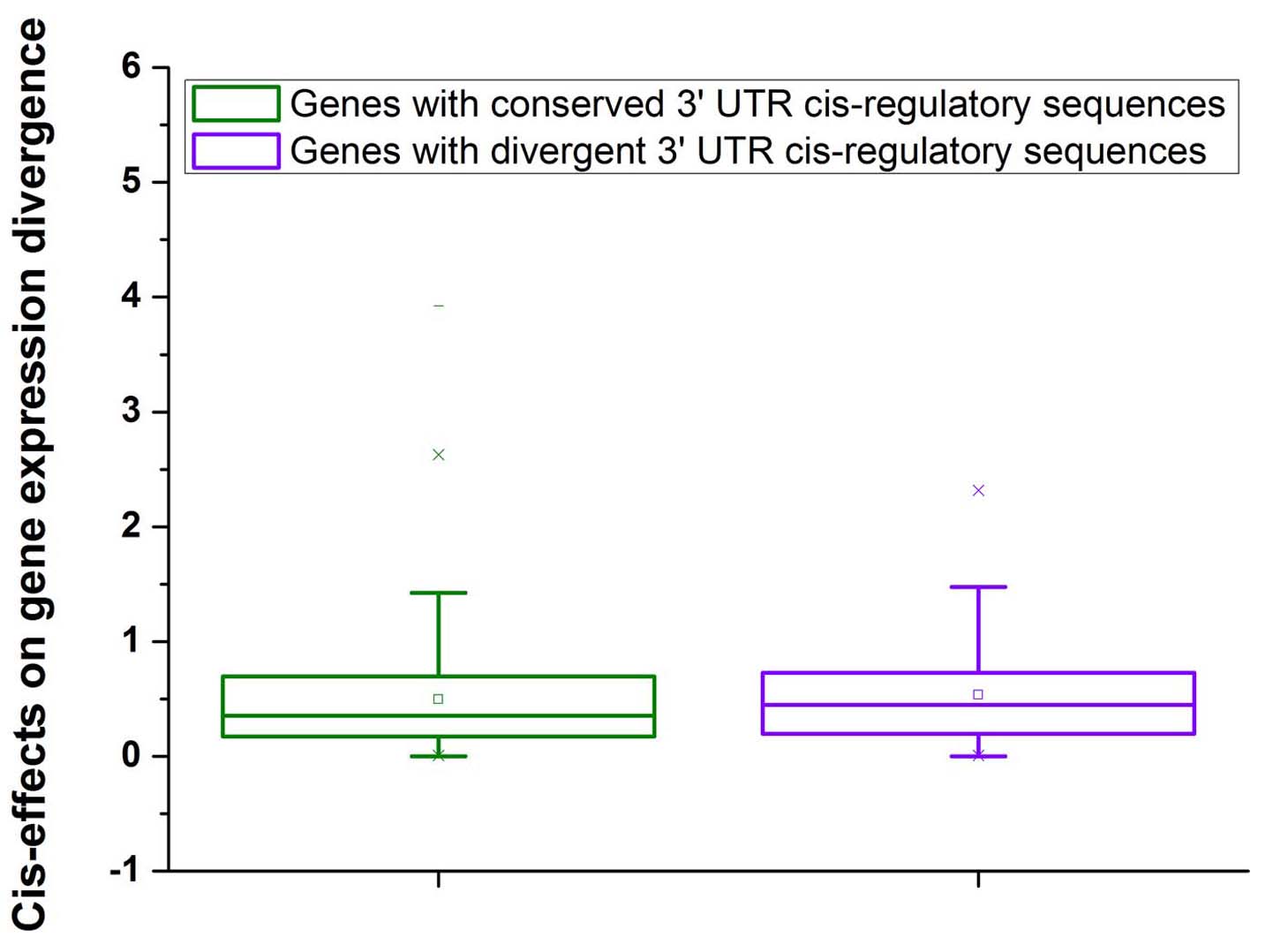

Supplement: Figure S2 — The relationship between changes in primary nucleotide sequences of 3′ UTR and gene expression divergence. Box plot of average values that correspond to levels of cis-effects on gene expression divergence are shown for genes whose 3′ UTR cis-regulatory sequences are less conserved (divergent) and genes with conserved 3′ UTR cis-regulatory sequences. Values were normalized using the function zscore in Matlab, such that their means are zero and standard deviations are one. (JPG) [file pcbi.1002275.s002.jpg]

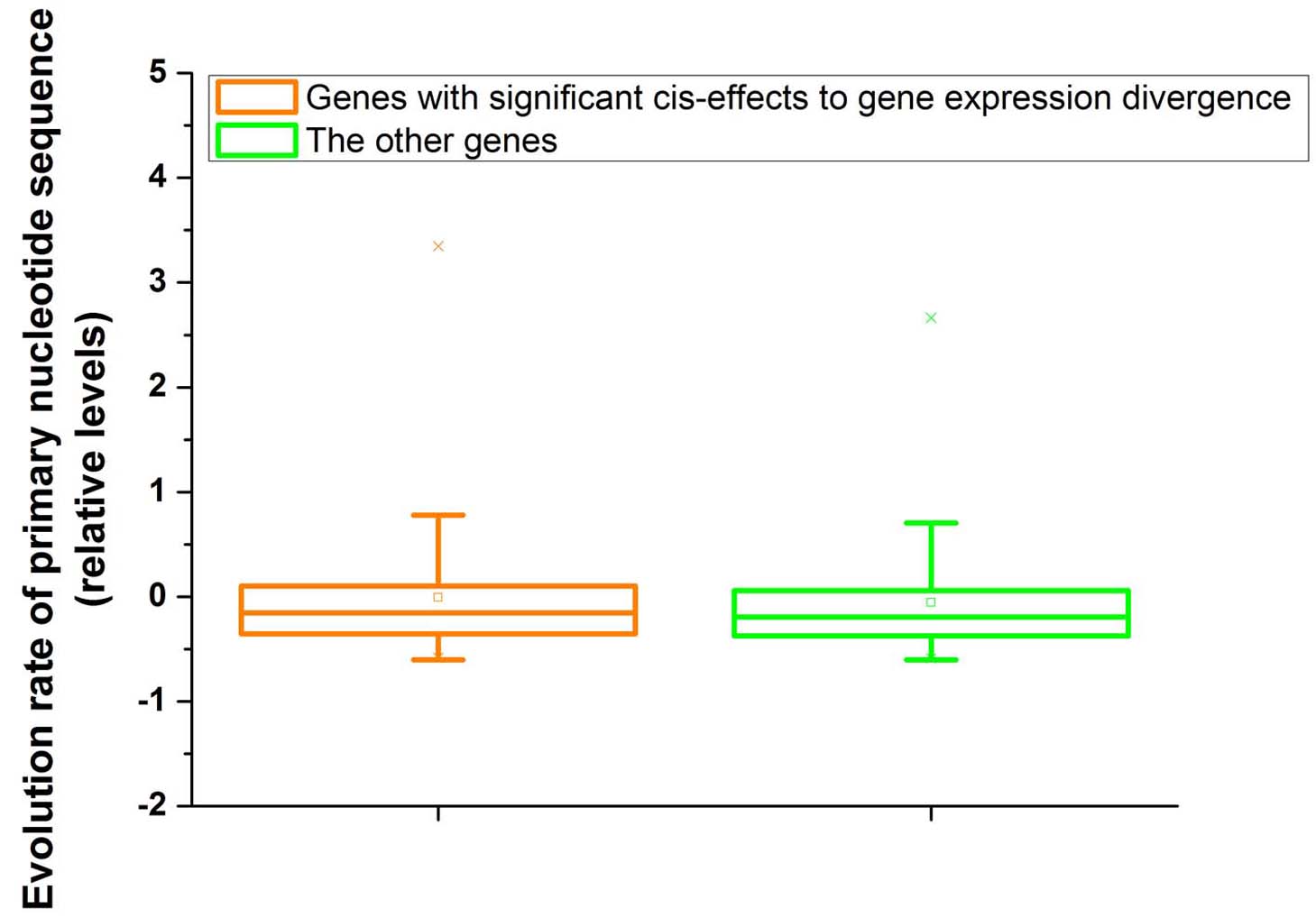

Supplement: Figure S3 — The relationship between changes in primary nucleotide sequences of coding regions and gene expression divergence. Box plot of average values that correspond to gene sequence evolutionary rates are shown for genes with significant cis-effects to gene expression divergence and the other genes. Values were normalized using the function zscore in Matlab, such that their means are zero and standard deviations are one. We performed the global alignment on orthologous gene sequences between S. cerevisiae and S. paradoxus, and used the rate of nonsynonymous substitutions (Ka) normalized by the rate of synonymous substitutions (Ks) as a measure of gene sequence evolutionary rate. (JPG) [file pcbi.1002275.s003.jpg]

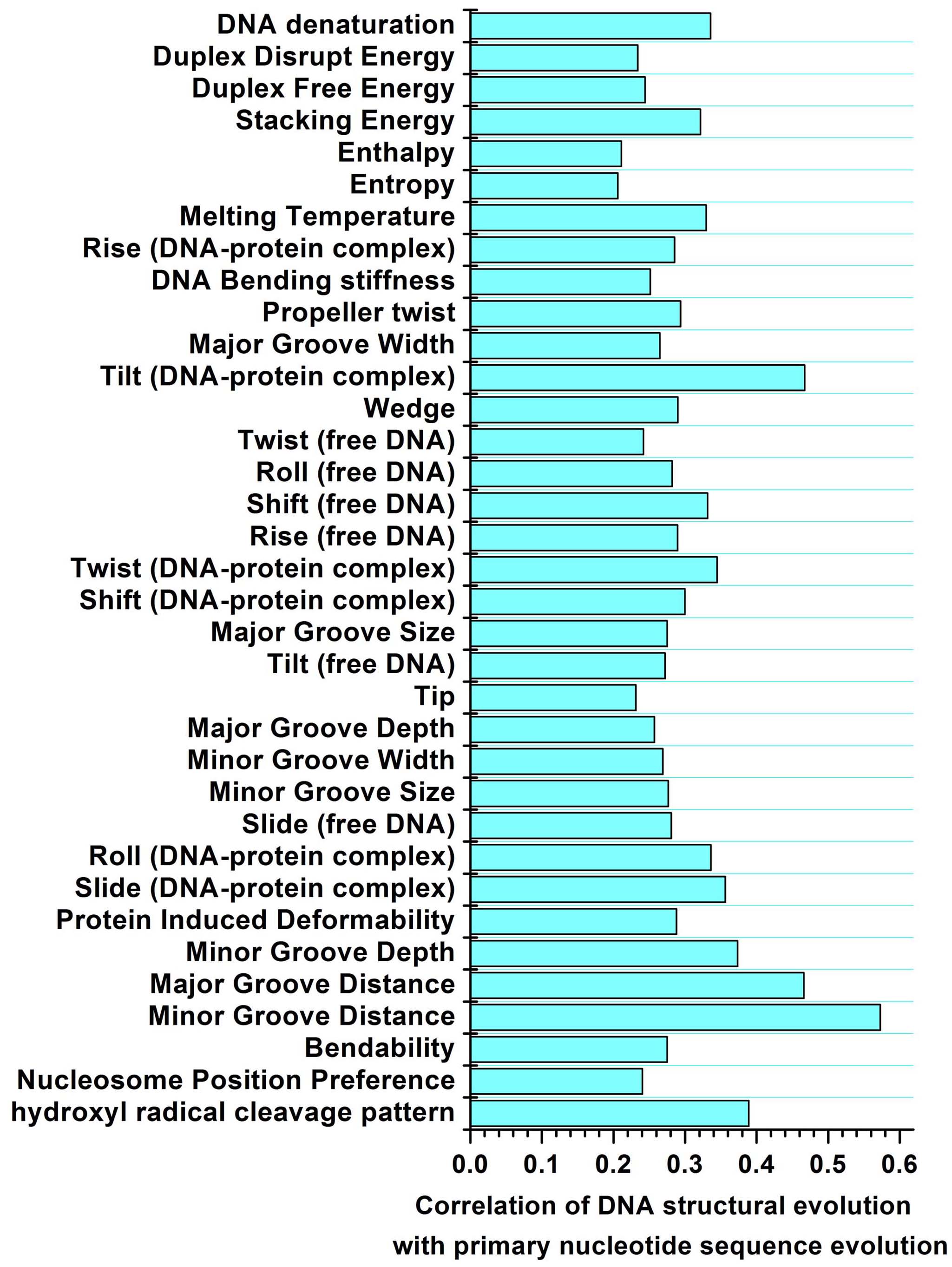

Supplement: Figure S4 — The correlation of DNA structural evolution rates with primary nucleotide sequence evolution rate. For each pair of orthologous genes between S. cerevisiae and S. paradoxus, we calculated gene sequence evolutionary rates and DNA structural evolution rate for each of the 35 DNA structural scales. The Pearson correlation coefficient between sequence evolutionary rates and structural evolution rates is shown for each of the 35 structural scales. (JPG) [file pcbi.1002275.s004.jpg]

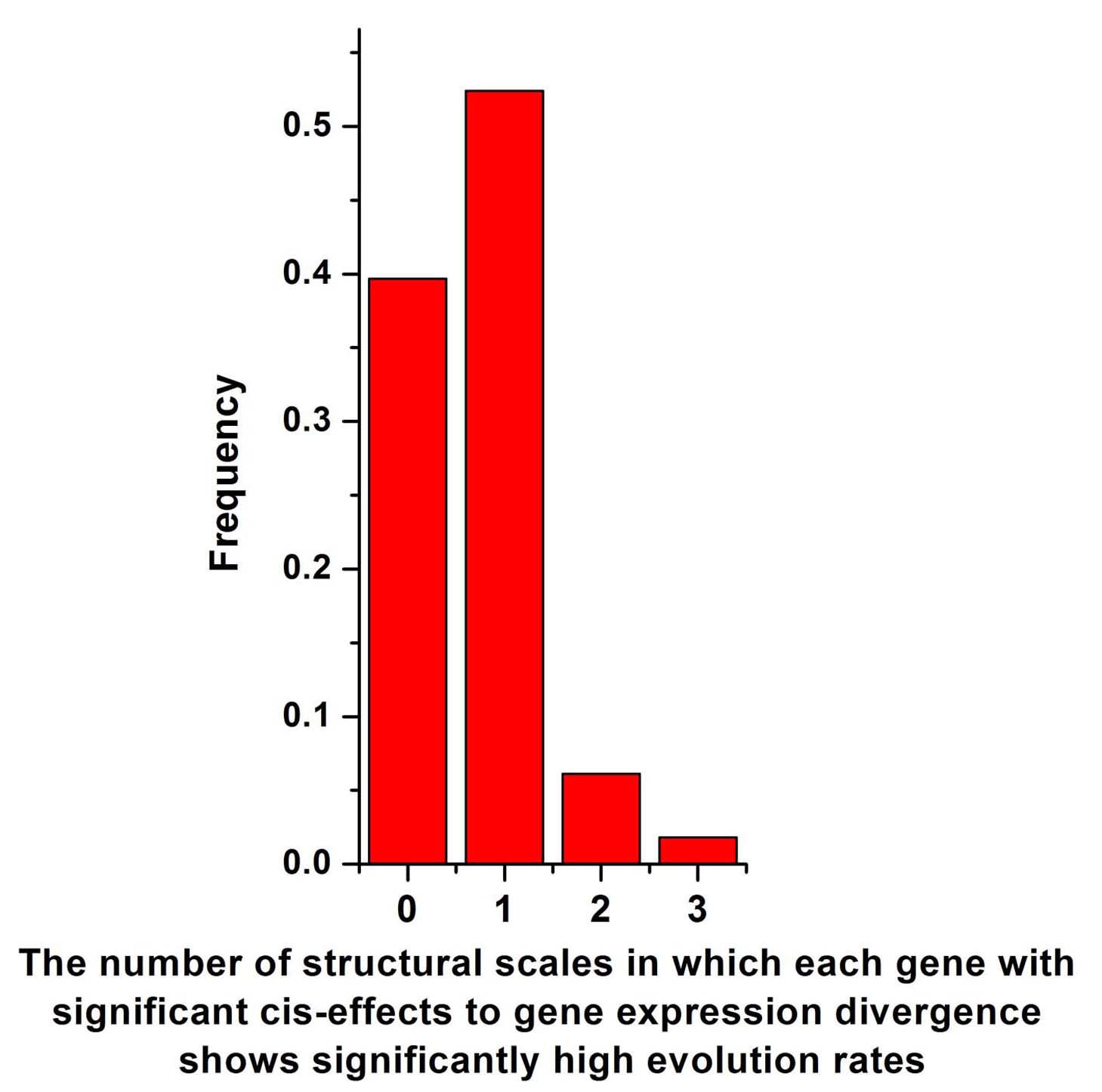

Supplement: Figure S5 — The number of structural scales in which each of the genes with significant cis-effects to gene expression divergence shows significantly high evolution rates (, ). The distribution of the numbers is shown. (JPG) [file pcbi.1002275.s005.jpg]

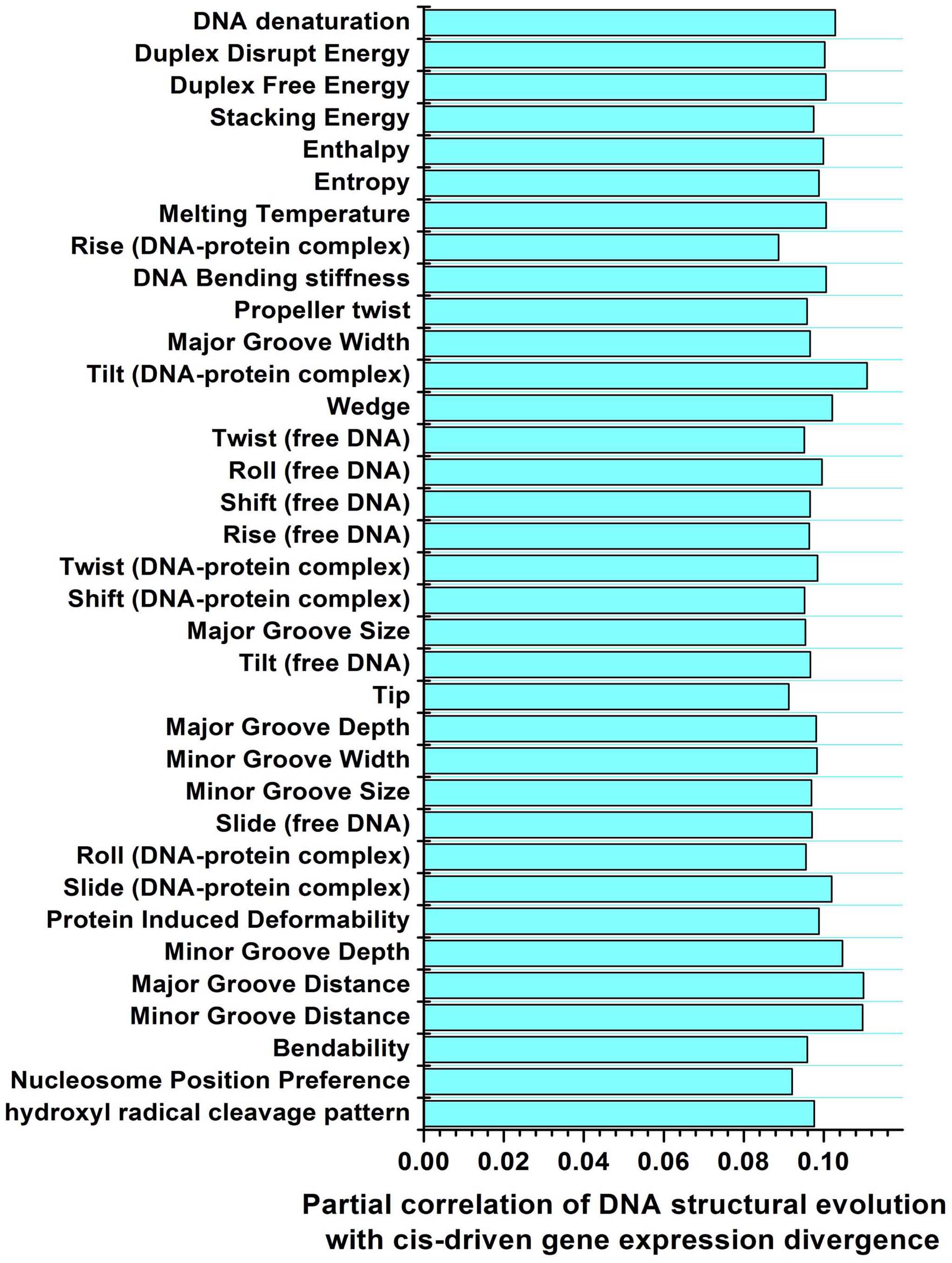

Supplement: Figure S6 — Partial correlation of DNA structural evolution rate with cis-driven gene expression divergence is shown for each of the 35 DNA structural scales when controlling primary nucleotide sequence evolution rates. Each bar represents the resulting partial correlation coefficients. (JPG) [file pcbi.1002275.s006.jpg]

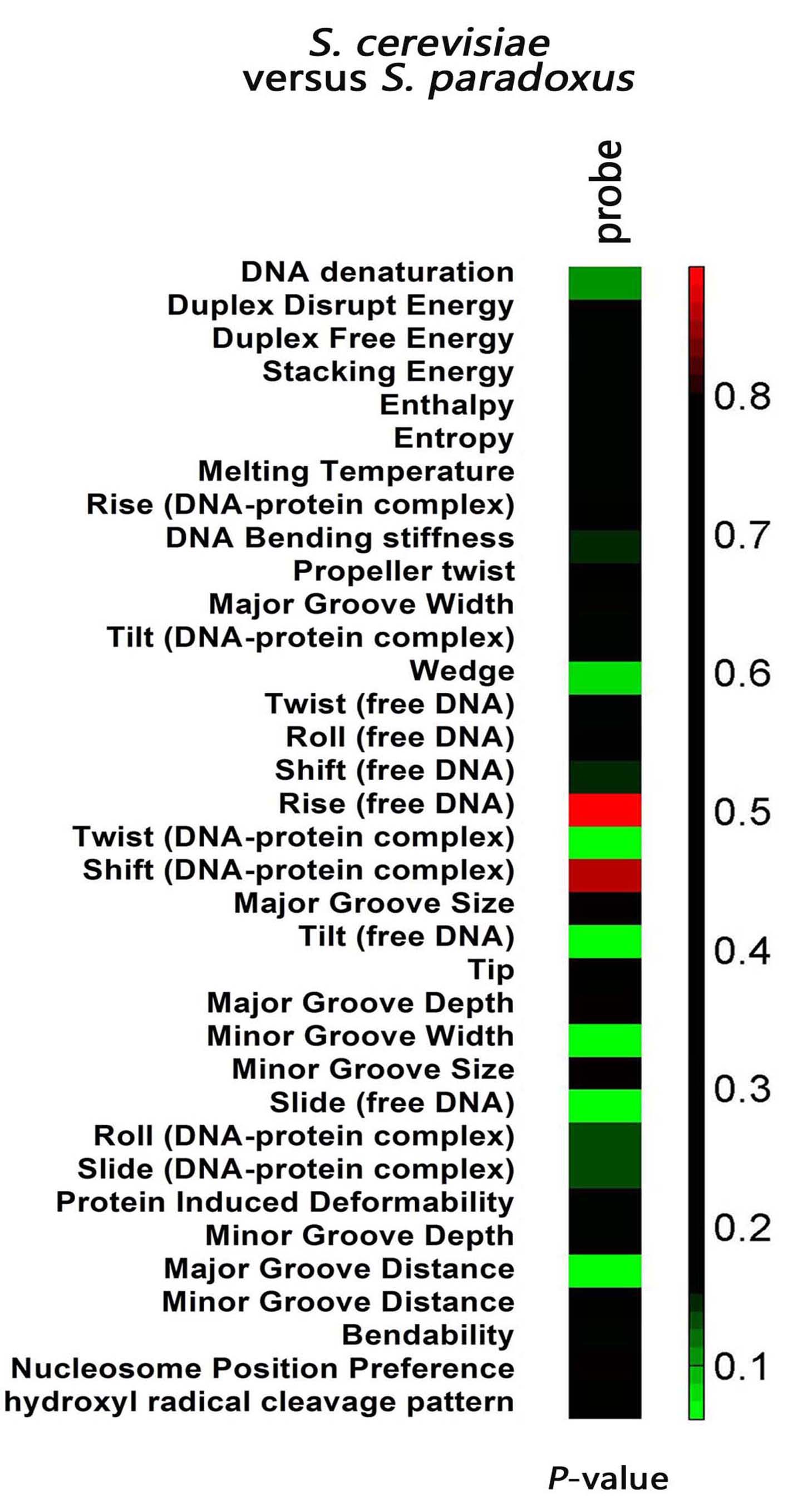

Supplement: Figure S7 — The relationship of cis-driven gene expression divergence between S.cerevisiae and S. paradoxus with DNA structural evolution in microarray probe regions. We compared the difference in evolution rates of 35 DNA structural scales in microarray probe regions between genes with significant cis-effects to gene expression divergence and the other genes. P-values were calculated through Mann-Whitney U-test, and are shown for the 35 DNA structural scales. Red (green) indicates high (low) P-values that evaluate the difference. (JPG) [file pcbi.1002275.s007.jpg]

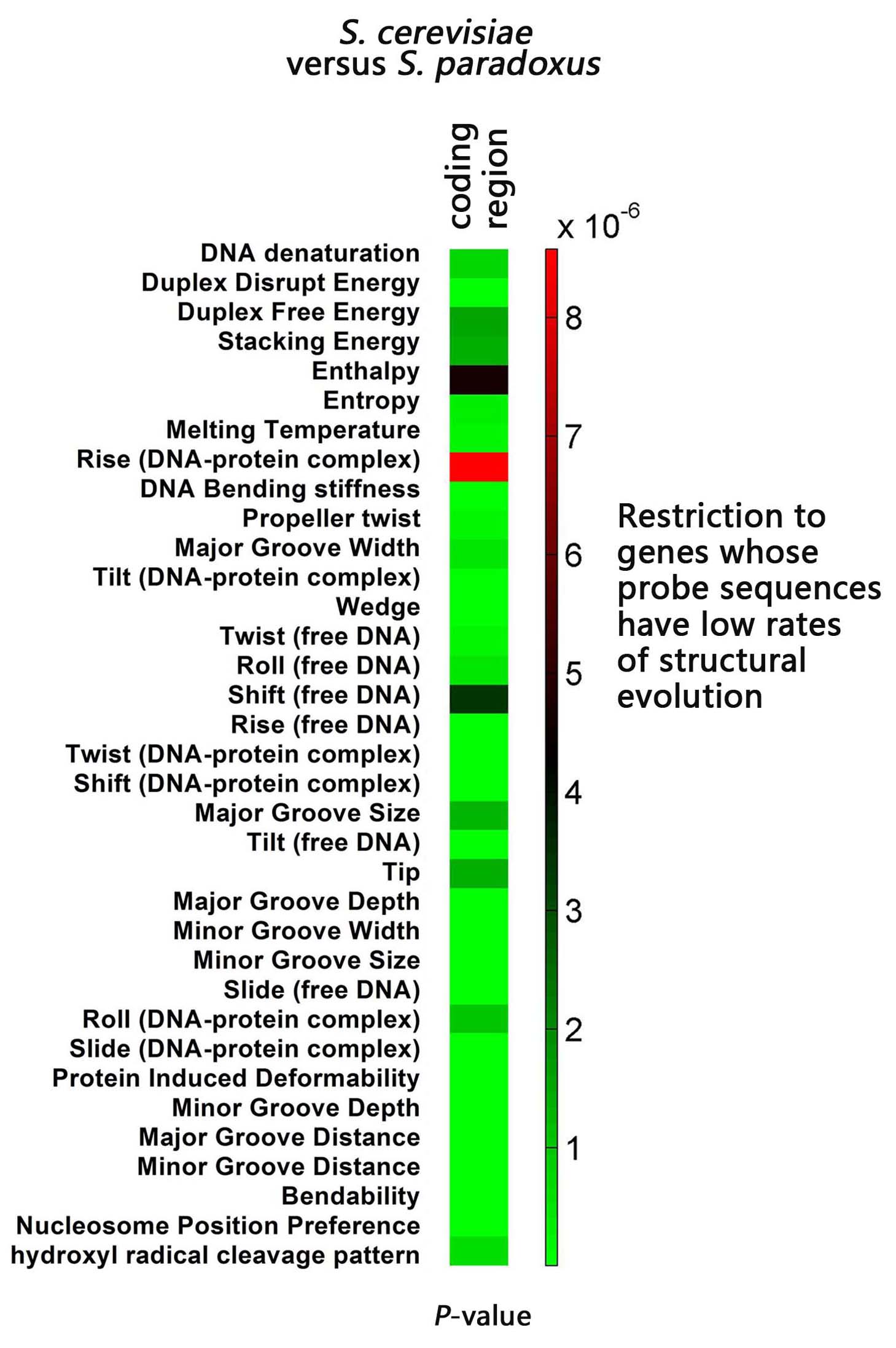

Supplement: Figure S8 — The relationship of cis-driven gene expression divergence between S. cerevisiae and S. paradoxus with DNA structural evolution when restricting analysis to genes whose probe sequences have low structural evolution rates (the 50% percentile). For each of the 35 DNA structural scales, we excluded genes whose probe sequences have high structural evolution rates (the 50% percentile), and compared the difference in DNA structural evolution rate between genes with significant cis-effects to gene expression divergence and the other genes in their coding regions. P-values were calculated through Mann-Whitney U-test, and are shown for the 35 DNA structural scales. Red (green) indicates high (low) P-values that evaluate the difference. (JPG) [file pcbi.1002275.s008.jpg]

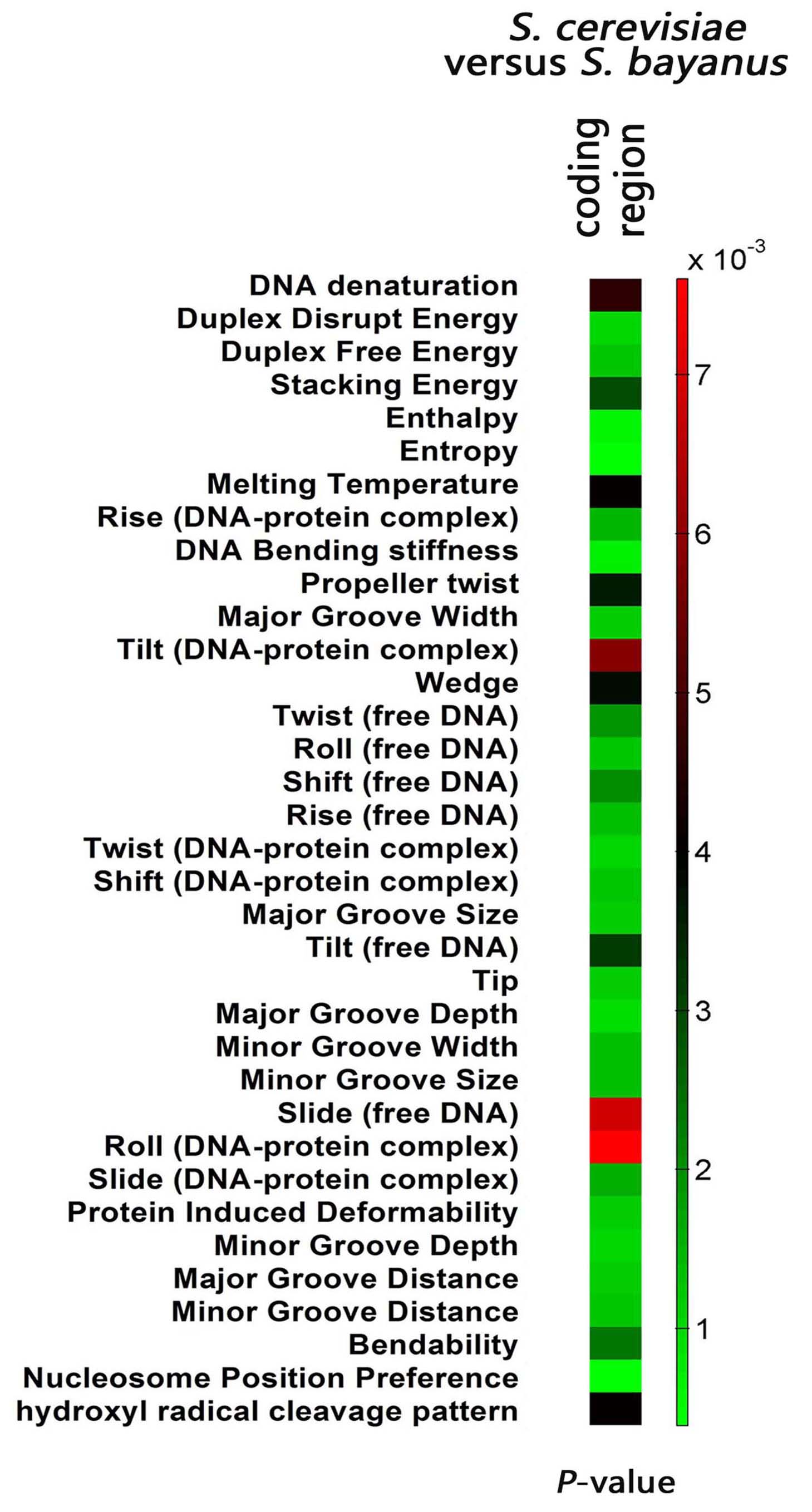

Supplement: Figure S9 — The relationship of cis-driven gene expression divergence between S. cerevisiae and S. bayanus with DNA structural evolution. We compared the difference in evolution rates of 35 DNA structural scales between genes with significant cis-effects to gene expression divergence and the other genes in their coding regions. P-values were calculated through Mann-Whitney U-test, and are shown for the 35 DNA structural scales. Red (green) indicates high (low) P-values that evaluate the difference. (JPG) [file pcbi.1002275.s009.jpg]

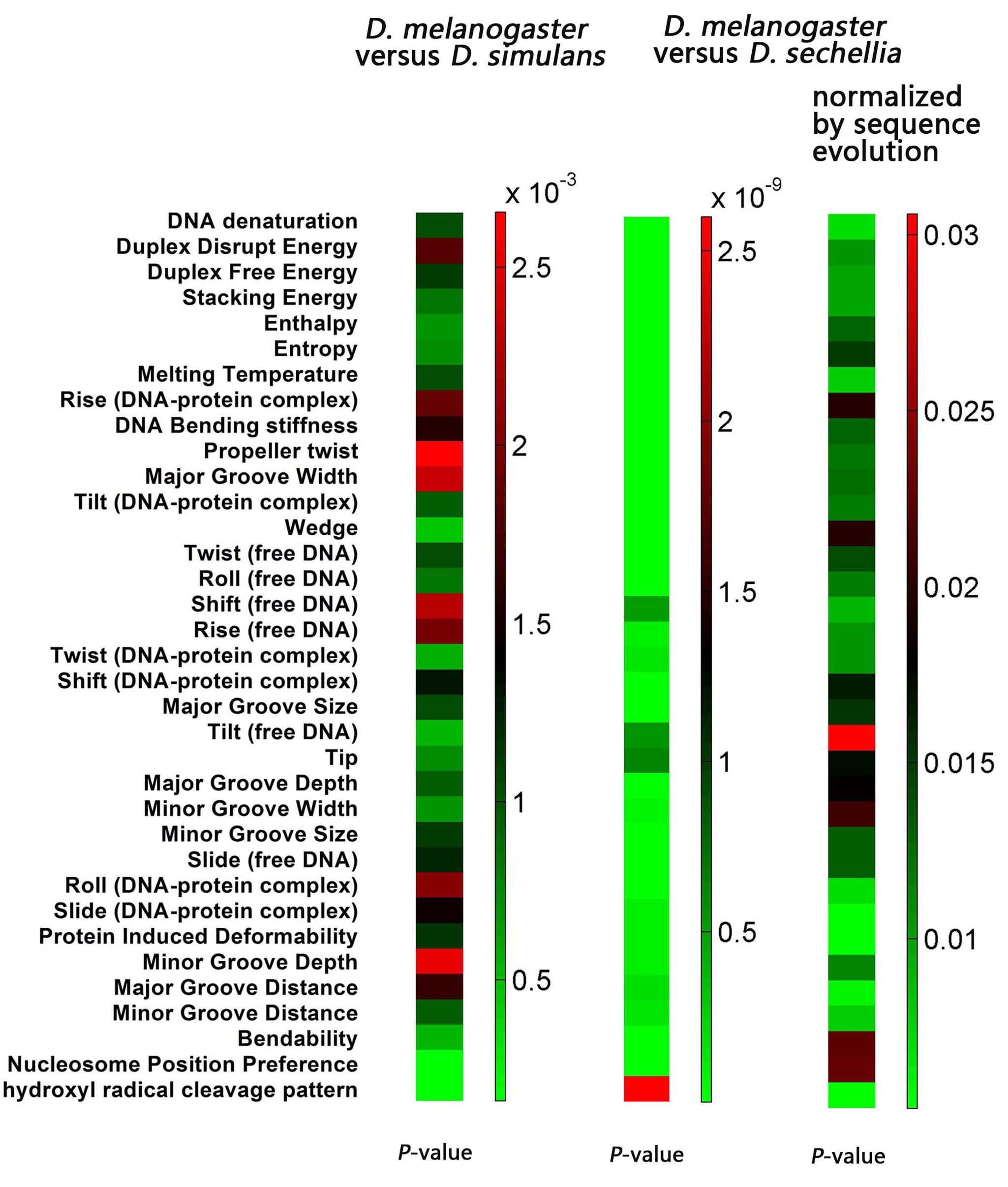

Supplement: Figure S10 — The relationship of cis-driven gene expression divergence in Drosophila species with DNA structural evolution. We compared the difference in evolution rates of 35 DNA structural scales between genes with significant cis-effects to gene expression divergence and the other genes in their coding regions. Comparison was also performed after normalizing DNA structural evolution rates by gene sequence evolution rates. P-values were calculated through Mann-Whitney U-test, and are shown for the 35 DNA structural scales. Red (green) indicates high (low) P-values that evaluate the difference. (JPG) [file pcbi.1002275.s010.jpg]

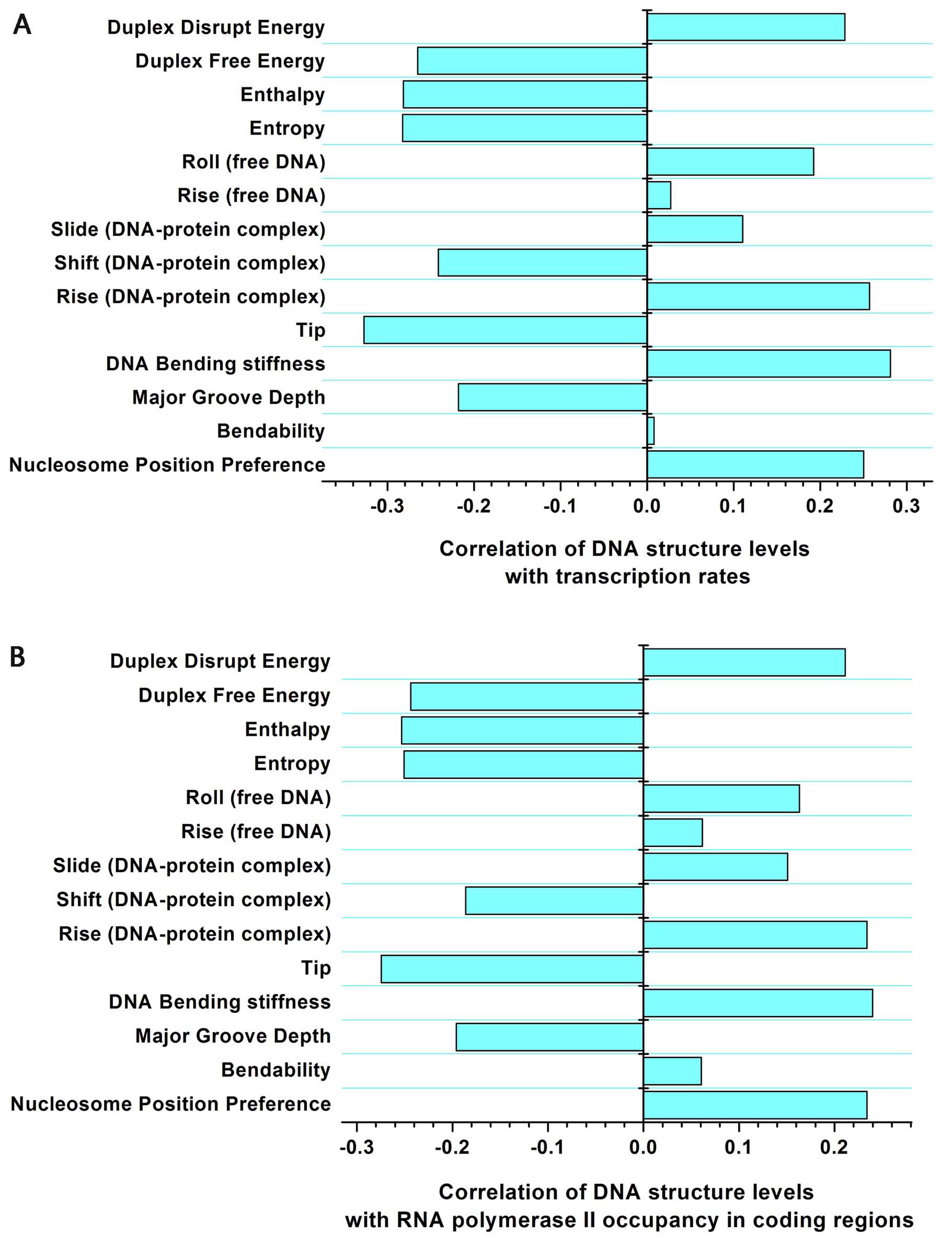

Supplement: Figure S11 — The correlation of DNA structural levels in coding regions with transcription rates and RNA polymerase II occupancy in coding regions. Each bar represents the resulting Pearson correlation coefficients for the 14 DNA structural scales. 12 out of the 14 scales show significant correlation with these two features (,). (JPG) [file pcbi.1002275.s011.jpg]
